# Efficient genome editing of differentiated renal epithelial cells

**DOI:** 10.1007/s00424-016-1924-4

**Published:** 2016-12-16

**Authors:** Alexis Hofherr, Tilman Busch, Nora Huber, Andreas Nold, Albert Bohn, Amandine Viau, Frank Bienaimé, E. Wolfgang Kuehn, Sebastian J. Arnold, Michael Köttgen

**Affiliations:** 1 Renal Division, Department of Medicine, Faculty of Medicine, University of Freiburg, Hugstetter Straße 55, 79106 Freiburg, Germany; 2 Spemann Graduate School of Biology and Medicine (SGBM), University of Freiburg, Freiburg, Germany; 3 Faculty of Biology, University of Freiburg, Freiburg, Germany; 4 Institute of Experimental and Clinical Pharmacology and Toxicology, Faculty of Medicine, University of Freiburg, Freiburg, Germany; 5 BIOSS Centre of Biological Signalling Studies, Albert-Ludwigs-University, Freiburg, Germany

**Keywords:** CRISPR, TALEN, MDCK, mIMCD3, PKD1, PKD2

## Abstract

Recent advances in genome editing technologies have enabled the rapid and precise manipulation of genomes, including the targeted introduction, alteration, and removal of genomic sequences. However, respective methods have been described mainly in non-differentiated or haploid cell types. Genome editing of well-differentiated renal epithelial cells has been hampered by a range of technological issues, including optimal design, efficient expression of multiple genome editing constructs, attainable mutation rates, and best screening strategies. Here, we present an easily implementable workflow for the rapid generation of targeted heterozygous and homozygous genomic sequence alterations in renal cells using transcription activator-like effector nucleases (TALENs) and the clustered regularly interspaced short palindromic repeat (CRISPR) system. We demonstrate the versatility of established protocols by generating novel cellular models for studying autosomal dominant polycystic kidney disease (ADPKD). Furthermore, we show that cell culture-validated genetic modifications can be readily applied to mouse embryonic stem cells (mESCs) for the generation of corresponding mouse models. The described procedure for efficient genome editing can be applied to any cell type to study physiological and pathophysiological functions in the context of precisely engineered genotypes.

**Electronic supplementary material:**

The online version of this article (doi:10.1007/s00424-016-1924-4) contains supplementary material, which is available to authorized users.

## Introduction

Analyses of epithelial physiology in genetically tractable model organisms have provided important biological insights. However, the complex interaction of multiple cell types and finite experimental resolution of specific cellular functions within tissues have made complementary cell culture-based approaches desirable. Wild-type renal cells have been characterized extensively, but considerable limitations concerning the genetic tractability of cultured cells have confounded molecular studies: the majority of isolated primary cells are heterogenous and have a finite replicative capacity; the generation of immortalized, differentiated renal epithelial cells from patients or mouse models has proven difficult; murine embryonic fibroblasts (MEFs) from genetically modified mice lack the epithelial characteristics of renal tubular cells; and the physiological relevance of heterologous expression systems has been questioned [9]. We therefore reasoned that the genetic manipulation of endogenously expressed proteins in differentiated renal epithelial cell lines may accelerate reaching novel insights into renal function. Indeed, genome edited renal epithelial cells have been successfully used to study renal epithelial physiology [e.g. 26, 29]. However, the practical implementation of required genome engineering technologies has been challenging for many laboratories. Thus, we provide a step-by-step protocol for efficient genome editing of differentiated renal epithelial and pluripotent cell types using TALEN [3, 5, 18] and CRISPR [7, 14, 17, 24] technology to generate targeted alleles within a short time-frame of 10 weeks at reasonable costs.

## Methods

Genome editing of MDCK, mIMCD3, and mES cells—See Supplementary Methods for step-by-step protocols.

### Molecular biology

Mouse *Pkd1* and human *PKD2* cDNA have been described previously [11]. All DNA constructs were validated by Sanger sequencing. Oligonucleotides for genotyping PCRs are listed in Supplementary Table 3 and Supplementary Table 4.

### Cell transduction

Constitutive gene expression was achieved by pLXSN-mediated (Clontech) retroviral transduction.

### RNA isolation and reverse transcription polymerase chain reaction

mRNA of a confluent 35 mm cell culture dish was isolated (RNeasy Plus Mini Kit, Qiagen) and reversely transcribed to complementary DNA (One Step RT-PCR Kit, Qiagen) according to the manufacturer’s protocols [1, 6]. Oligonucleotides for RT-PCRs are listed in Supplementary Table 5.

### Antibodies

Mouse anti-beta-Actin (Clone AC-15; Sigma-Aldrich), goat anti-TRPP2 (G-20; Santa Cruz Biotechnology), mouse anti-Polycystin-1 (7E12; Santa Cruz Biotechnology), chicken anti-GFP (ab13970; Abcam), mouse anti-Flag M2 (clone M2; Sigma-Aldrich), rabbit anti-V5 epitope tag (Merck Millipore), and mouse anti-V5-Tag (Clone SV5-Pk1; Bio-Rad) antibodies were obtained commercially. Mouse anti-TRPP2^698–799^ antibodies have been described previously [12, 13]. Western blot detection was performed using an anti-mouse (Dako) horse-radish peroxidase-coupled secondary antibody. Antigens were visualized by immunofluorescence using secondary goat anti-chicken Alexa Fluor 488 (Thermo Fisher Scientific).

### Protein isolation, SDS-PAGE, Western blot and ECL detection

Cells were harvested 5 days after epithelial confluency. Proteins were isolated and processed as described previously [12, 13]. Chemiluminescence was detected by either a 16-bit ChemoCam system (Intas) or by Super RX film (Fujifilm).

### Immunofluorescence staining of cells

Indirect immunofluorescence staining of cells has been described previously [12]. Cells were fixed by paraformaldehyde (Electron Microscopy Sciences). Primary (GFP, 1:2′000) and secondary antibodies (1:1′000) were diluted in PBS. F-actin was stained by Alexa Fluor 568 Phalloidin (A12380, Thermo Fisher Scientific). DNA was probed using DAPI (Sigma-Aldrich). Bright-field images were recorded using an Axio Observer microscope (Zeiss).

## Results

**Fig. 1:**
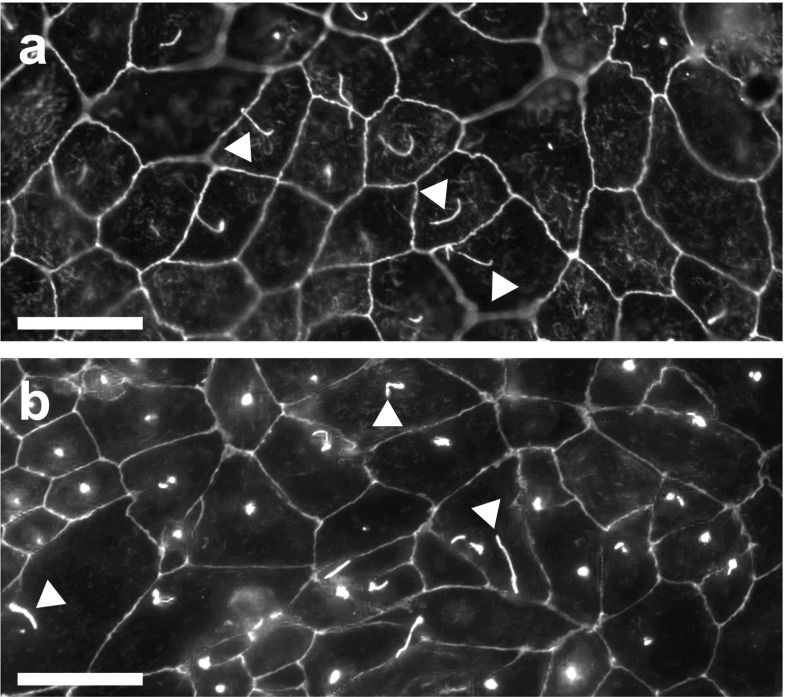
MDCK and mIMCD3 cells are highly differentiated renal epithelial cells. **a** MDCK and **b** mIMCD3 cells stained for acetylated tubulin (aTub) to mark primary cilia (*white arrows*) and ZO1 to visualize tight junctions as markers for epithelial cell differentiation. *Scale bars* = 20 μm

To date, American Type Culture Collection lists 21 mammalian renal epithelial cell lines (www.atcc.org). Of those, Madin-Darby Canine Kidney (MDCK) cells and mouse Inner Medullary Collecting Duct 3 (mIMCD3) cells are most widely used to study renal epithelial biology (≥ 5′000 entries on www.ncbi.nlm.nih.gov) [8, 22]. Both cell lines are phenotypically stable and retain core epithelial characteristics, including apico-basolateral polarity, directed epithelial transport, primary cilia, and well-defined cell junctions in 2D and 3D culture (Fig. 1) [4, 10]. Furthermore, cells proliferate rapidly and are suitable for biochemical, physiological, and imaging-based experiments as well as high throughput screening.

### Genome editing in renal epithelial cells

**Fig. 2:**
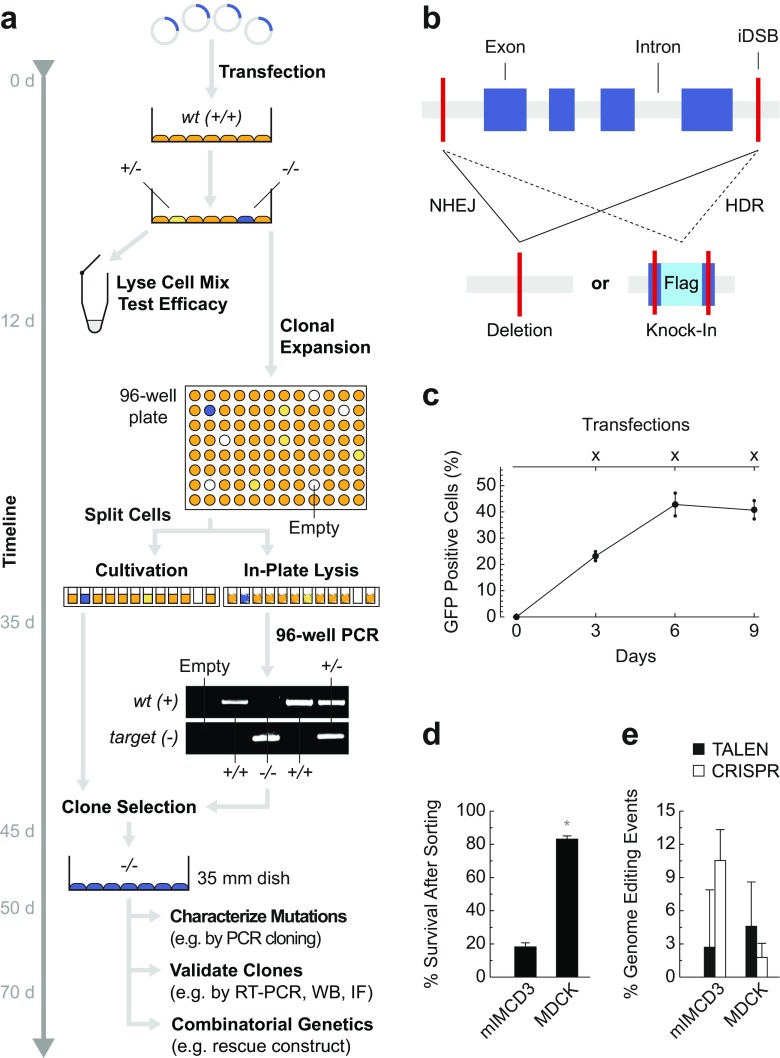
Genome editing of MDCK and mIMCD3 renal epithelial cells. **a** Here, we provide a practical guide to generate genetically modified renal epithelial cell culture models. Genome editing requires transfection of programmable sequence-specific nucleases to induce DNA double strand breaks (iDSB) [15, 21]. After PCR-based testing for genome editing efficacy, cells are singularized in 96-well plates and clones are PCR-screened for targeted alleles. Targeted clones (*−/−*) are selected and validated by independent methods. *d* days, *WB* Western blot, *IF* immunofluorescence. See Supplementary Methods for a step-by-step protocol. **b** Nuclease-induced DSBs are repaired by non-homologous end joining (NHEJ) or homology directed repair (HDR). Both mechanisms can be exploited for genome editing [15, 21]. For example, if two DSBs are induced, error prone NHEJ may skip the middle piece causing a deletion; or, if two DSBs are induced and a targeting vector is provided, precise HDR may incorporate exogenous sequence elements (here, a Flag epitope tag for biochemical protein detection and isolation) into the genome. **c** Repeated transfection of cells increases the proportion of transfected cells significantly. MDCK cells were repeatedly nucleofected with GFP and fluorescent cells were counted using an automated cell counter (*N* = 3; for each *N* we analyzed ≥2′000 cells). **d** In contrast to MDCK cells, mIMCD3 cells are more sensitive to the transfection and sorting procedure requiring higher cell numbers a priori (*N* = 5; wells evaluated after single cell sorting / *N* ≥ 1′000). **e** To evaluate genome editing efficacy in MDCK and mIMCD3 cells we targeted three genes per cell line with both, TALEN and CRISPR technology. Target alleles for TALENs and CRISPR were similar, but not identical, due to different genomic binding site requirements. We aimed for large deletions (≥10 kb) or HDR-mediated integrations of targeting vectors. Correctly targeted heterozygous or homozygous clones were counted. Minor NHEJ-mediated sequence changes were not considered. Significant genome editing events were observed in 3.19% of MDCK and 6.62% of mIMCD3 cells (*N* = 3 per cell line and genome editing technology; for each *N* we analyzed between 90 and 1′101 clones). Data are presented as means with s.e.m.

To take advantage of well-characterized cell lines, we have developed a widely applicable workflow for the efficient generation of targeted alleles using genome editing technology (Fig. 2a). During protocol optimization we have identified several critical steps that facilitate gene targeting significantly. Those include enhanced genome editing construct design for rapid and reliable testing of editing efficacy, repeated transfections in intervals to increase targeting efficiencies, simplified design of targeting vectors with or without selection cassettes for homologous recombination, scalable clonal selection procedures, and streamlined screening approaches for positively targeted clones (Fig. 2a–e and Supplementary Fig. 1a–f). Details for the different experimental steps are discussed in the Supplementary Methods. Our protocols provide a means-tested basis to achieve genetically modified cell lines based on non-homologous end joining (NHEJ) and homology directed repair (HDR) (Fig. 2a–e, Supplementary Fig. 1a–f, and Supplementary Methods).

### Generation of cell culture models for ADPKD

**Fig. 3:**
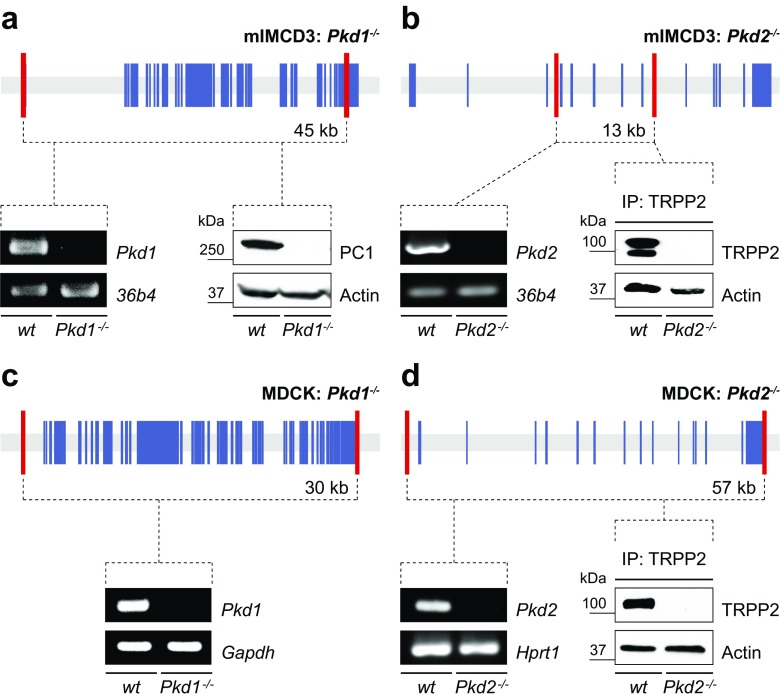
Genome editing-based generation of ADPKD cell culture models. *Pkd1* (**a**, **c**) and *Pkd2* (**b**, **d**) were deleted in mIMCD3 (**a**, **b**) and MDCK (**c**, **d**) cells. Loss of ADPKD genes was validated by genome sequencing (Supplementary Table 1), non-existence of respective mRNA and/or absence of polycystin proteins. Shown are ADPKD gene structures with respective exons (*blue*), location of iDSB (*red*), size of genomic deletion, RT-PCR, and Western blot experiments validating genomic knock-out of ADPKD genes. We were not able to detect endogenous PC1 protein in MDCK cells. *36b4*, *Gapdh*, and *Hprt1* served as positive controls for RT-PCR; beta-Actin as loading control for Western blot. *IP* immunoprecipitation

**Fig. 4:**
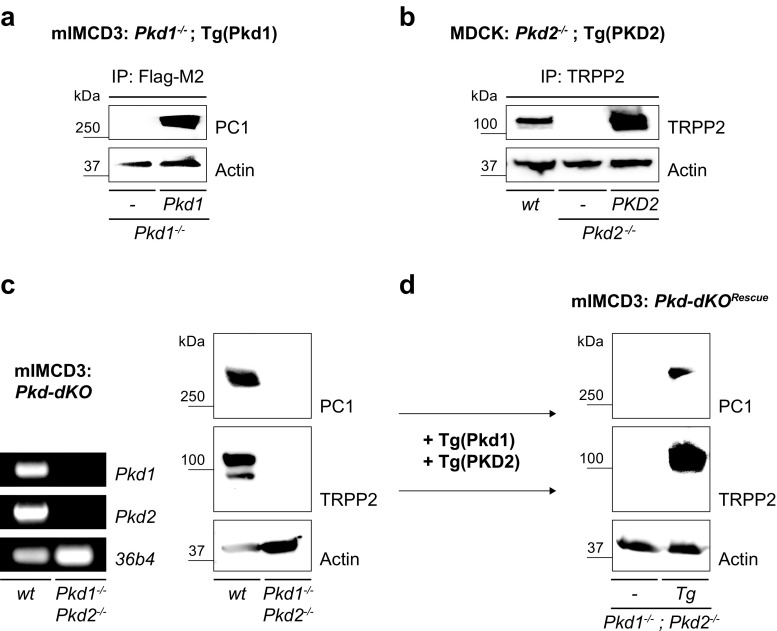
Combinatorial genetics complement the study of ADPKD in vitro. In the study of biological processes, it is often desirable to be able to genetically recombine specific sequence elements or manipulate multiple genes simultaneously. We used genome editing-based combinatorial genetics to generate ADPKD cell culture models beyond loss of ADPKD gene function. Proper controls are essential to help ensure success in every experiment; therefore, we genetically rescued *Pkd1* (Fig. 2a) and *Pkd2* (Fig. 2d) deficiency using retrovirus-mediated transduction to generate **a** mIMCD3 *Pkd1* ^*−/−*^; Tg(Pkd1) and **b** MDCK *Pkd2* ^*−/−*^; Tg(PKD2) cells. Polycystin expression in these cell lines was validated by Western blot. Rescue cell lines may help to ensure that observed effects are due to knock-out of PKD genes. Knock-out of both ADPKD genes, on the other hand, fully inactivates the polycystin signaling module providing a maximal ADPKD model. **c** Loss of PKD genes in mIMCD3 cells (*Pkd-dKO*) was shown by RT-PCR and Western blot. (**d**) Virus transduction-mediated rescue of polycystin expression was demonstrated by Western blot (*Pkd-dKO* ^Rescue^). *36b4* served as positive controls for RT-PCR; beta-Actin as loading control for Western blot. *IP* immunoprecipitation, *Tg* transgene

**Fig. 5:**
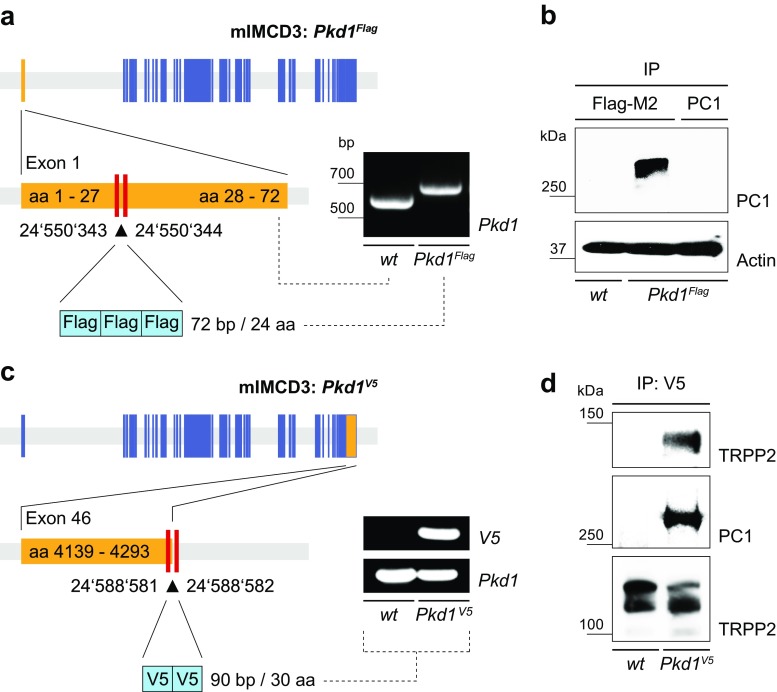
Targeted epitope tagging of endogenous PC1 facilitates biochemical and mass spectrometry-based analysis. PC1 protein detection and purification is particularly difficult problem in ADPKD research severely constraining biochemical analysis of polycystin signaling. To overcome this issue, we introduced well-established exogenous epitope tags into the endogenous PC1 protein. **a** Introduction of three Flag epitope tags into the mouse *Pkd1* genomic locus by homologous recombination (mIMCD3 *Pkd1* ^Flag^) was tested by respective size shift of genomic PCR (Supplementary Fig. 1e). **b** Endogenous PC1^Flag^ can be immunoprecipitated efficiently with anti-Flag M2 antibodies facilitating mass spectrometry-based analysis (Supplementary Table 6). On the other hand, IP of PC1 using a specific antibody was inefficient. **c** Similarly, introduction of two V5 epitope tags into the mouse *Pkd1* genomic locus (mIMCD3 *Pkd1* ^V5^) was validated by PCR (Supplementary Fig. 1f). A V5 epitope tag-independent *Pkd1* PCR served as positive control (oligonucleotides mPkd1-Flag_1 and mPkd1-Flag_2). **d** Endogenous PC1^V5^ can be precipitated and detected with rabbit anti-V5 and mouse anti-V5 antibodies, respectively. Native TRPP2 co-precipitates with PC1^V5^. *IP* immunoprecipitation

The loss-of-function phenotypes of the ADPKD genes, *polycystic kidney disease 1* (*PKD1*) and *PKD2*, suggest that both genes are part of an epithelial morphogenetic program to maintain kidney structure [23, 27]. Yet, the molecular function of the respective proteins, Polycystin-1 (PC1) and transient receptor potential channel Polycystin-2 (TRPP2), has remained elusive [2]. To demonstrate the versatility of our genome editing approach, we have established multiple novel cell lines for the study of ADPKD by introducing targeted mutations into *PKD1* and *PKD2* (Supplementary Table 1–4): (1) deletions of PKD genes (Fig. 3a–d); (2) rescues of polycystin expression (Fig. 4a, b); (3) generation of cell lines incorporating multiple allelic features (Fig. 4c, d); and (4) introduction of epitope tags into the *Pkd1* genomic locus by homologous recombination (Fig. 5a–d). The introduction of epitope tags into the *PKD1* locus exemplifies the power of genome editing. Affinity purification of PC1 is notoriously difficult (Fig. 5b). HA- and Myc-tagged *PKD1* alleles have been described previously, but purification of sufficient amounts of native PC1 protein for mass spectrometry-based proteomics has not been reported [30]. Here, we show that Flag epitope-tagged native PC1 can be purified with high affinity facilitating reliable detection by mass spectrometry (Fig. 5b and Supplementary Table 6). Furthermore, we confirm that endogenous PC1 and TRPP2 co-immunoprecipitate using a V5 epitope-tagged PC1 (Fig. 5d) [20, 28].

### Translation of validated alleles to mouse models

**Fig. 6:**
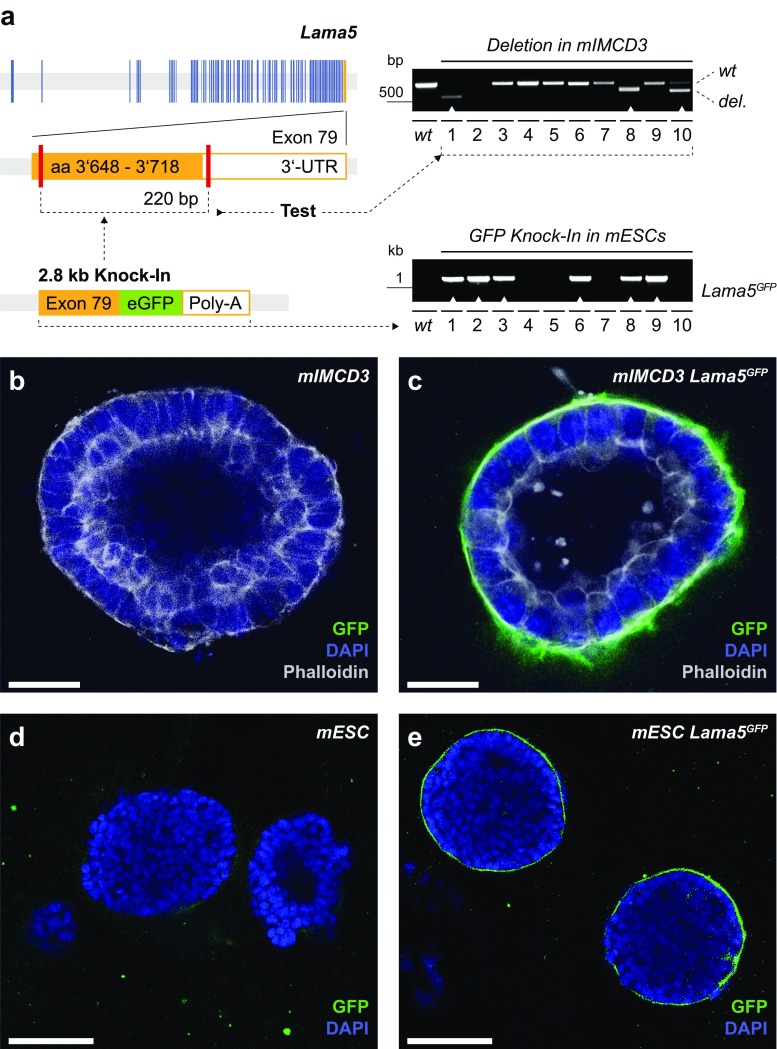
Testing of functional alleles in mIMCD3 cells and transfer into mESCs**. a** Gene structure of mouse *Lama5* with exons as *blue* bars. TALENs were directed to induce two double strand breaks (iDSB, *red bars*) in the last coding exon (# 79) of the *Lama5* gene leading to a deletion of 220 bp in proximity to the terminator TGA-codon. TALEN-efficiency was tested in mIMCD3 cells generating a 568 bp deletion band (wild-type = 788 bp). Using a 2.8 kb targeting construct mIMCD3 and mES cells were targeted by homologous recombination to genetically fuse an eGFP cDNA to the 3′-end of the *Lama5* coding sequence (Supplementary Figure 2a-d). Due to the C57BL/6J mouse origin of mIMCD3 cells, the same TALENs and targeting vector can be applied to mESCs. **b**, **c** Wild-type (**b**) and targeted (**c**) mIMCD3 cells were grown in 3D culture to form spheroids. Targeted *Lama5* ^GFP^ mIMCD3 cells express a green fluorescent basal lamina. **d**, **e** Similarly, embryoid bodies, derived from genetically engineered mESCs express a green fluorescent basal lamina. These cells may be injected into mouse blastocysts to generate chimeric mice. *Scale bars* = 100 μm

We have shown that genetically engineered cells provide valuable tools to complement in vivo models. Equally useful, however, is the inverse approach: utilization of tissue-specific cell culture models for functional validation of genetically engineered alleles before generation of respective mouse models. Even though the generation of genetically engineered mice is continuously improving, it is still an expensive and hugely time-consuming procedure. Genome editing has been used to generate mutant alleles in mice [e.g. 25, 31]. Here, we propose that testing of alleles in differentiated cells may help to facilitate the generation of complex genetic mouse models. One example of this application is the addition of fluorescent tags to proteins of interest. Ideally, the function of such engineered fusion proteins should be validated before generation of the respective mouse line. As a proof of principle, we have generated a fluorescently tagged allele of the basal lamina component, *Laminin subunit alpha 5* (*Lama5*). Basal lamina biogenesis is a core epithelial function, but its visualization in vivo has been difficult. The generation of fluorescent laminin fusion proteins showing sufficient brightness and proper localization to the basal lamina has been particularly challenging. To visualize basal lamina, we genetically fused eGFP to the 3′-end of the *Lama5* coding sequence (Fig. 6a and Supplementary Figure 2a–d). Resulting mIMCD3 cells express a fluorescent LAMA5 (LAMA5^GFP^) that can be studied in 3D culture (Fig. 6b, c) [10]. Obviously, a complementing in vivo system is likely to provide additional insights. The C57BL/6J mouse origin of mIMCD3 cells, i.e. their isogenic status to commonly used mESCs, is key to the direct transferability into mouse models [22]. Accordingly, we applied the mIMCD3-validated genome editing constructs to the *Lama5* locus in mESCs. Comparable targeting efficiencies (mIMCD3 = 55%; mESCs = 50%) suggest that genomic alterations in mESCs can be readily tested in mIMCD3 cells and successively used for the establishment of novel mouse alleles (Fig. 6a–e).

## Discussion

Programmable sequence-specific nucleases are highly efficient genome editing tools in MDCK, mIMCD3, and mES cells. Thus, TALEN and CRISPR technology may complement RNAi-based approaches, which are limited to inhibition of post-transcriptional gene expression. The modular nature of TALEN and CRISPR systems facilitates additional applications other than genome editing, including selective labeling of nucleotide sequences, transcriptional regulation, or alteration of epigenetic DNA modifications [15, 21]. Similar to genome editing all these applications are based on the sequence-specific targeting of enzymes by the nucleotide-binding TALEN or CRISPR construct. Therefore, although their sequence specificity has been shown to be fairly high—especially in comparison to RNAi—experimental designs based on TALEN or CRISPR constructs have to control for putative off-target effects [16, 19]. For genome editing-mediated generation of cellular loss-of-function models, we suggest the combination of a complete gene excision with subsequent gene rescue (Fig. 4a–d). NHEJ-mediated short sequence alterations are commonly used, but may be difficult to interpret due to alternative splicing or stop codon read-through, which may result in residual protein function. In contrast, complete gene excision generates definite null alleles with complete loss of protein function (Fig. 3 and Supplementary Fig. 1c). Rescue of the gene excision by reintroduction of the respective coding sequence can accurately distinguish, whether an observed phenotype is gene-dependent or caused by deletion of additional regulatory sequences in the mutant background. HDR-mediated genome editing, furthermore, facilitates the study of physiological functions of endogenous proteins (Fig. 5a–d and Fig. 6a–e). Targeted mutation or introduction of tags, for example, may elucidate the molecular mechanisms of native proteins.

## Conclusions

Here, we present an easily applicable workflow for efficient genetic manipulation of differentiated renal epithelial cell lines. The development of genetically engineered MDCK and mIMCD3 cells with isogenic controls provides novel versatile tools to study renal biology under well-controlled conditions. Importantly, the described protocols can be readily adjusted to other cell types, including mESCs for the efficient generation of novel mouse models. In addition, we show that complex genetic alterations can be tested in differentiated cell types before the corresponding mouse strains are established. Taken together, we anticipate that described protocols will contribute to a broader use of genome editing technology for functional studies of renal epithelial cells as well as other cell types in health and disease.

## Electronic supplementary material

ESM 1 (PDF 785 kb)

ESM 2 (PDF 609 kb)

ESM 3 (PDF 230 kb)
