# Supplementary figures and images for: Efficient genome editing of differentiated renal epithelial cells
Source: Pflugers Arch. 2016 Dec 16;469(2):303–11. doi: 10.1007/s00424-016-1924-4 (PMC5222933; doi:10.1007/s00424-016-1924-4)

# Supplementary Figure 1

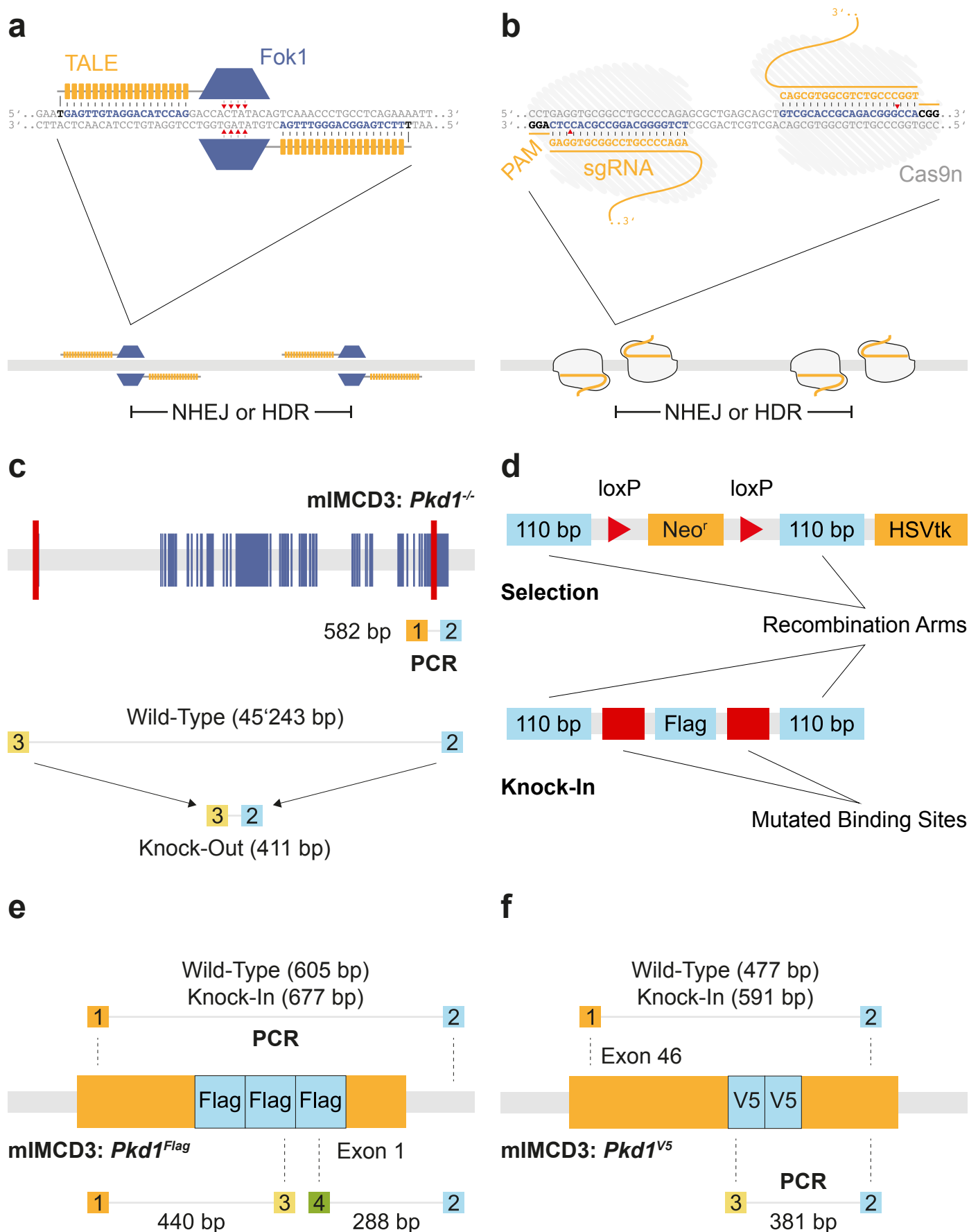

# Supplementary Figure 2

a

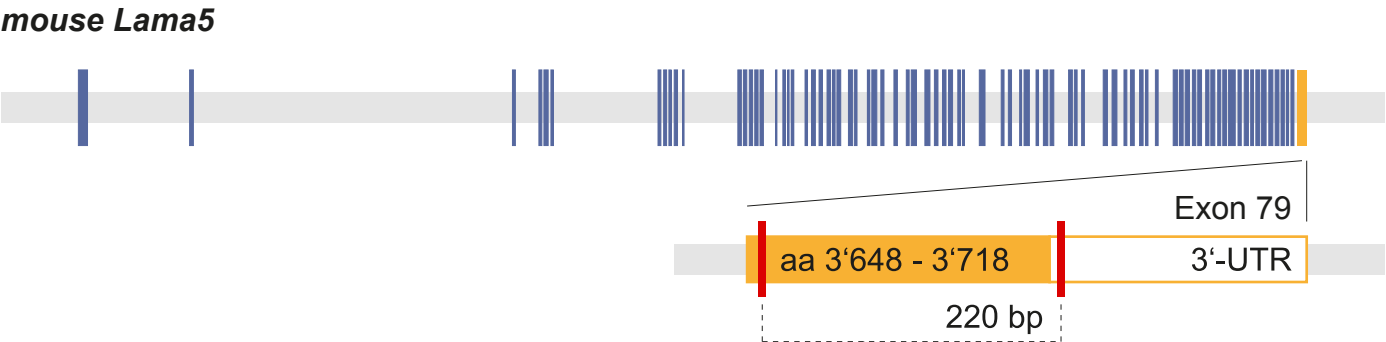

b

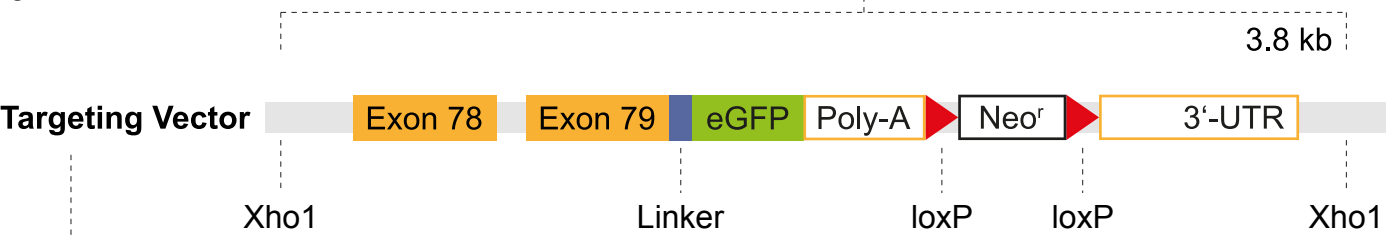

c

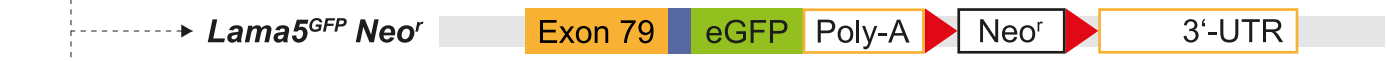

d

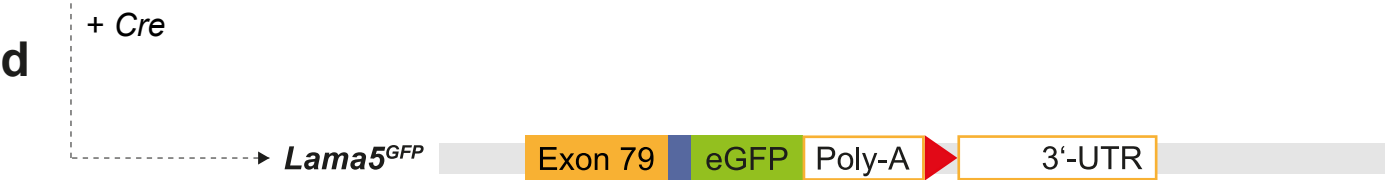

Supplement: Supplementary file 1 — (PDF 785 kb) [file 424_2016_1924_MOESM1_ESM.pdf]
