# Supplementary material for: Efficient genome editing of differentiated renal epithelial cells
Source: Pflugers Arch. 2016 Dec 16;469(2):303–11. doi: 10.1007/s00424-016-1924-4 (PMC5222933; doi:10.1007/s00424-016-1924-4)
Supplement: Supplementary file 2 — (PDF 609 kb) [file 424_2016_1924_MOESM2_ESM.pdf]

Supplementary Table 1 - Cell lines.

**Mouse Inner Medullary Collecting Duct 3 cell lines (mIMCD3)**				
**Name**	**Gene/s**	**Reference Alleles**	**Clones$^{-/-}$**	**Clones$^{+/-}$**
Pkd1	*Pkd1*	∆ Chr. 17: 24‘550‘055 - 24‘594‘963 /<br>∆ Chr. 17: 24‘550‘106 - 24‘594‘903	3	
Pkd1^{Rescue}	*Pkd1*	∆ Chr. 17: 24‘550‘055 - 24‘594‘963 /<br>∆ Chr. 17: 24‘550‘106 - 24‘594‘903 ;<br>Tg(Pkd1)	1	
Pkd1^{Flag}	*Pkd1*	Tg(3×Flag) after Chr. 17: 24‘550‘343	2	5
Pkd1^{V5}	*Pkd1*	Tg(2×V5) after Chr. 17: 24‘588‘581		3
Pkd2	*Pkd2*	∆ Chr. 5: 104‘478‘281 - 104‘490‘878	4	
Pkd2^{Rescue}	*Pkd2*	∆ Chr. 5: 104‘478‘281 - 104‘490‘878 ;<br>Tg(PKD2)	1	
Pkd-dKO	*Pkd1* + *Pkd2*	∆ Chr. 5: 104‘478‘281 - 104‘490‘878 ;<br>∆ Chr. 17: 24‘550‘042 - 24‘594‘890 /<br>∆ Chr. 17: 24‘550‘106 - 24‘594‘903	3	
Pkd-dKO^{Rescue}	*Pkd1* + *Pkd2*	∆ Chr. 5: 104‘478‘281 - 104‘490‘878 ;<br>∆ Chr. 17: 24‘550‘042 - 24‘594‘890 /<br>∆ Chr. 17: 24‘550‘106 - 24‘594‘903 ;<br>Tg(Pkd1) ;<br>Tg(PKD2)	1	
Lama5^{GFP}	*Lama5*	Tg(GFP) after Chr. 2: 180‘176‘550		3

**Madin-Darby Canine Kidney cell lines (MDCK)**				
**Name**	**Gene/s**	**Reference Alleles**	**Clones$^{-/-}$**	**Clones$^{+/-}$**
Pkd1	*Pkd1*	∆ Chr. 6: 38‘836‘147 - 38‘865‘462	5	1
Pkd1^{Rescue}	*Pkd1*	∆ Chr. 6: 38‘836‘147 - 38‘865‘462 ;<br>Tg(Pkd1)	1	
Pkd2	*Pkd2*	∆ Chr. 32: 11’381’232 - 11’438’424	2	2
Pkd2^{Rescue}	*Pkd2*	∆ Chr. 32: 11’381’232 - 11’438’424 ;<br>Tg(PKD2)	1	

Supplementary Table 2 - TALEN and sgRNA binding sites.

Mouse Inner Medullary Collecting Duct 3 cells (mIMCD3)			
**Target**	**Type**	**Name**	**Binding site**
Pkd1	CRISPR	mPkd1_sgRNA_1	TCTGGGGCAGGCCGCACCTC
		mPkd1_sgRNA_2	GTCGCACCGCAGACGGGCCA
		mPkd1_sgRNA_3	AGAGCTCGCCACGCAAGGCG
		mPkd1_sgRNA_4	ACCCCAGGACTATGAGATGG
***Pkd1**^{Flag}*	CRISPR	mPkd1^{Flag}_sgRNA_1	CGAGCCACAGGCCCAGGCCT
		mPkd1^{Flag}_sgRNA_2	GAGACCCTGGGCGCGGCTGC
		mPkd1^{Flag}_sgRNA_3	GCGCAGGGCCGCAAAAGCAG
		mPkd1^{Flag}_sgRNA_4	GCGTCAATTGCTCCGGCCGC
***Pkd1**^{V5}*	CRISPR	mPkd1^{V5}_sgRNA_1	AATAACAAGGTCCACCCCAG
		mPkd1^{V5}_sgRNA_2	CCTAATAACAAGGTCCACCC
Pkd2	TALEN	mPkd2_TALEN_1	CAGTAAATGCAGAGAGGA
		mPkd2_TALEN_2	ACATGTAGAGTCAGTCCA
		mPkd2_TALEN_3	CTCTCATGCTTGATCACC
		mPkd2_TALEN_4	TCTGCTTTTCATAGGTTA
Lama5	TALEN	mLama5_TALEN_1	TCATAGAAAGTGTAGGCA
		mLama5_TALEN_2	TTGGCATTGAGGGTTCCC
		mLama5_TALEN_3	CGGTAGGCAGGGAGCTCT
		mLama5_TALEN_4	CTGTATTTTCAGAGTCTT
**Madin-Darby Canine Kidney cells (MDCK)**			
**Target**	**Type**	**Name**	**Binding site**
Pkd1	TALEN	cPkd1_TALEN_1	GCTGGTTGCCGTGAGACC
		cPkd1_TALEN_2	GCTTCACCCTGCAGACCT
		cPkd1_TALEN_3	GGCCACAGTTACAGCCTT
		cPkd1_TALEN_4	TATTTGACTTTGTCGGGT
Pkd2	TALEN	cPkd2_TALEN_1	TTTAAGGTAACTAAGTTA
		cPkd2_TALEN_2	TGGATTCTGGCCTATTCT
		cPkd2_TALEN_3	TGGTAAGTTGCCAATCAA
		cPkd2_TALEN_4	TCCCATCAGATCAAAGTG

Supplementary Table 3 - Genotyping of mIMCD3 cell lines.

Mouse Inner Medullary Collecting Duct 3 cell lines (mIMCD3)			
**Feature**	**Name**	**Oligonucleotide**	**Product (bp)**
Pkd1	mPkd1_1	AGATGGCCATCCTGGTAGGT	582
	mPkd1_2	CTAGGTGGGAGCACAACTGG	
Pkd1$^{-/-}$	mPkd1_3	CCACCTACAGCCAACTTGGA	45‘243 (wt)<br>300 + 411 (-/-)
	mPkd1_2	CTAGGTGGGAGCACAACTGG	
Pkd1^{Flag}	mPkd1-Flag_1	CCACCTACAGCCAACTTGGA	605 (wt)<br>677 (Flag)
	mPkd1-Flag_2	CCCAGTCATGATGCTCTAGCC	
	mPkd1-Flag_1	CCACCTACAGCCAACTTGGA	440
	mPkd1-Flag_3	GTCCTTGTCATCGTCGTCCT	
	mPkd1-Flag_4	GACTACAAGGACGACGATGA	288
	mPkd1-Flag_2	CCCAGTCATGATGCTCTAGCC	
Pkd1^{V5}	mPkd1-V5_1	GAGCAACAACTCCAGAGCCT	477 (wt)<br>591 (V5)
	mPkd1-V5_2	GCTGGGTACTCACTTGGTCC	
	mPkd1-V5_2	GCTGGGTACTCACTTGGTCC	381
	mPkd1-V5_3	GGTGGTGGCAAACCCATA	
Pkd2	mPkd2_1	TTAGTAAGCGCGTGTCCA	476
	mPkd2_2	ACTGAGGCAAACTGCCAT	
Pkd2$^{-/-}$	mPkd2_3	TATGGCAGGTCCCTGTGG	12‘895 (wt)<br>296 (-/-)
	mPkd2_2	ACTGAGGCAAACTGCCAT	
Lama5	mLama5_1	TGGAGGTAGACACACAGAGCAACC	788 (wt)<br>568 (deletion)
	mLama5_2	CCGTCCCCAAGTTGGAATTG	
Lama5^{GFP}	mLama5-GFP_1	GCAGTGAGGGAGTTGTCACATTAGG	1‘138
	mLama5-GFP_2	AACTTCAGGGTCAGCTTGCCGTAG	
	mLama5-GFP_3	AGACTGCCTTGGGAAAAGCG	1‘041
	mLama5-GFP_4	CCTTGCCAATGCAGACGTTC	

Supplementary Table 4 - Genotyping of MDCK cell lines.

Madin-Darby Canine Kidney cell lines (MDCK)			
**Feature**	**Name**	**Oligonucleotide**	**Product (bp)**
Pkd1	cPkd1_1	TGGAGCTGTTAGGTGTCC	1‘040
	cPkd1_2	CCACTGCCTGCCCTTCGA	
Pkd1$^{-/-}$	cPkd1_1	TGGAGCTGTTAGGTGTCC	30‘323 (wt)<br>1‘007 (-/-)
	cPkd1_3	CCCTGGAGGGCTCTCTGC	
Pkd2	cPkd2_1	ACAGGAGTCAGAGTGCTG	861
	cPkd2_2	ACCTGTGTGCCTTACCCA	
Pkd2$^{-/-}$	cPkd2_3	ACAAAGGATGCTGTCCGG	57‘725 (wt)<br>532 (-/-)
	cPkd2_2	ACCTGTGTGCCTTACCCA	

Supplementary Table 5 - Oligonucleotides for RT-PCR.

Mouse Inner Medullary Collecting Duct 3 cell lines (mIMCD3)			
**mRNA**	**Name**	**Oligonucleotide**	**Product (bp)**
36b4	RT_m36b4_1	CCGATCTGCAGACACACACT	91
	RT_m36b4_2	ACCCTGAAGTGCTCGACATC	
Pkd1	RT_mPkd1_1	GTGGAAAGCAGGTCGGAAG	236
	RT_mPkd1_2	TCGTCTCGTTCAGCACCAG	
Pkd2	RT_mPkd2_1	GTGGATGTACACAAGTGAGAAGGAGC	454
	RT_mPkd2_2	CACGACAATCACAACATCCAGACA	
**Madin-Darby Canine Kidney cell lines (MDCK)**			
**mRNA**	**Name**	**Oligonucleotide**	**Product (bp)**
Gapdh	RT_cGapdh_1	CATGTTTGTGATGGGCGTGAACCA	100
	RT_cGapdh_2	TTTGGCTAGAGGAGCCAAGCAGTT	
Hprt1	RT_cHprt_1	TCCATTCCTATGACTGTAG	189
	RT_cHprt_1	ATTATGCTCCTTGACCAA	
Pkd1	RT_cPkd1_1	TTCTGCTGGTCTCCTCCTGT	270
	RT_cPkd1_2	GCAGAAAGAGCTCCACCATC	
Pkd2	RT_cPkd2_1	CCAGCCGAGAGAAGTATC	142
	RT_cPkd2_2	AAGAAGAGTTGTGACATTATCC	

Supplementary Table 6 - Polycystin-1^{Flag} nano-LC-MS/MS peptide data.

Mouse Inner Medullary Collecting Duct 3 (mIMCD3) *Pkd1^{Flag}*		
**#**	**Unique PC1 Peptides**	**Position in PC1**
1	WLQTLGPSLR	55 - 64
2	EHQVHVVQSEATTCR	142 - 156
3	EAFLTAAEFSTQKLEEPAQMR	585 - 605
4	LEEPAQMR	598 - 605
5	LTPLLGLGPNPGLQHPGHYEVR	772 - 793
6	ATVGNSVSR	794 - 802
7	VLQGILVR	939 - 946
8	YSPMVEAGSDVAFR	947 - 960
9	SGIFPLALVLSSHVNK	1‘346 - 1‘361
10	VSGWNEVSR	1‘521 - 1‘529
11	TSMPSTTHTFAAPGLHLVR	1‘759 - 1‘777
12	VGDHLVNVQAENHVSHAQAQVR	1‘939 - 1‘960
13	VQGDSLVILSGR	2‘009 - 2‘020
14	FEAATSPSPR	2‘075 - 2‘084
15	NYLEAHVDLR	2‘163 - 2‘172
16	LVPIIEGGSYR	2‘252 - 2‘262
17	VWSDTQDLVLDGSK	2‘263 - 2‘276
18	GSSVVTIPLER	2‘315 - 2‘325
19	AQAVYEVSR	2‘371 - 2‘379
20	DAEDGGAPLVYALLLK	2‘496 - 2‘511
21	VLNEEPLTLAGEEIVALGK	2‘763 - 2‘781
22	VASMAFQTQTGTQIPIEQLAAER	2‘843 - 2‘865
23	ITYTVLNER	2‘916 - 2‘924
24	ISLEVLEGADHR	2‘955 - 2‘966
25	LLVAELQR	3‘240 - 3‘247
