# Supplementary material for: Efficient genome editing of differentiated renal epithelial cells
Source: Pflugers Arch. 2016 Dec 16;469(2):303–11. doi: 10.1007/s00424-016-1924-4 (PMC5222933; doi:10.1007/s00424-016-1924-4)
Supplement: Supplementary file 3 — (PDF 230 kb) [file 424_2016_1924_MOESM3_ESM.pdf]

Efficient genome editing of differentiated renal epithelial cells

Alexis Hofherr, MD, PhD[1, 2, 3, *], Tilman Busch, MD[1, *], Nora Huber[4], Andreas Nold[1], Albert Bohn[1], Amandine Viau, PhD[1], Frank Bienaimé, MD, PhD[1], E. Wolfgang Kuehn, MD[1], Sebastian J. Arnold, MD[4, 5] & Michael Köttgen, MD[1]

* Both authors contributed equally to this work.

Affiliations

[1] Renal Division, Department of Medicine, Faculty of Medicine, University of Freiburg, Hugstetter Straße 55, 79106 Freiburg, Germany

[2] Spemann Graduate School of Biology and Medicine (SGBM), University of Freiburg, Freiburg, Germany

[3] Faculty of Biology, University of Freiburg, Freiburg, Germany

[4] Institute of Experimental and Clinical Pharmacology and Toxicology, Faculty of Medicine, University of Freiburg, Freiburg, Germany

[5] BIOSS Centre of Biological Signalling Studies, Albert-Ludwigs-University, Freiburg, Germany

Correspondence

Alexis Hofherr (alexis.hofherr@uniklinik-freiburg.de)

Michael Köttgen (michael.koettgen@uniklinik-freiburg.de)

Telephone: +49-(0)761-270-63140 / Fax: +49-(0)761-270-63240

Supplementary Data

- Supplementary Methods
- Supplementary Figures 1-2
- Supplementary Table 1-6

Supplementary Methods

Reagents

Cell Culture

- Madin-Darby Canine Kidney (MDCK) cells (3)
- mouse Inner Medullary Collecting Duct 3 (mIMCD3) cells (ATCC, cat. no. CRL-2123) (10)
- CCE mouse embryonic stem cells (mESCs) (1)
- Dulbecco's Modified Eagle Medium (DMEM) high glucose with L-glutamine (Lonza, cat. no. BE12-604F)
- DMEM : Ham's F12 (1 : 1 Mix) with 15mM HEPES and L-glutamine (Lonza, cat. no. BE12-719F)
- Fetal bovine serum (FBS; Biochrom, cat. no. S0115)
- ESC certified FBS (Gibco, cat. no.16141-079)
- Glutamine 100× (Gibco, cat. no. 15030-024)
- Penicillin-Streptomycin (Sigma-Aldrich, cat. no. P0781)
- Phosphate Buffered Saline powder without Ca^{2+} / Mg^{2+} (PBS; Biochrom, cat. no. L1825)
- 0.25 % Trypsin-EDTA (Gibco, cat. no. 25200056)
- Dimethyl sulfoxide (DMSO; Sigma-Aldrich, cat. no. D2650)
- Geneticin (G418, 25'000 U / ml; Biochrom, cat. no. A2912)
- Ganciclovir (Sigma-Aldrich, cat. no. G2536)
- Mitomycin C (Sigma-Aldrich, cat. no. M4287)
- Leukemia Inhibitory Factor (LIF; ESGRO, Merck Milipore, cat. no. ESG1107)
- 2-Mercaptoethanol (Sigma-Aldrich, cat. no. M6250)
- Gelatine, from porcine skin (Sigma-Aldrich, cat. no. G1890)

Plasmids & Transfection

- Cell Line Nucleofector Kit L (Lonza, cat. no. VCA-1005)
- Cell Line Nucleofector Kit R (Lonza, cat. no. VCA-1001)
- pX330-U6-Chimeric-BB-CBh-hSpCas9 (Addgene, cat. no. 42230)
- pX335-U6-Chimeric-BB-CBh-hSpCas9n (D10A) (Addgene, cat. no. 42335)
- Golden Gate TALEN and TALE Effector Kit 2.0 (Addgene, cat. no. 1000000024)

Chemicals & Enzymes

- Proteinase K (Roche, cat. no. 03115879001)
- Potassium chloride (KCl; Merck cat. no. 104936)
- Magnesium chloride ($MgCl_2$; Merck cat. no. 105833)
- Igepal CA-630 (Sigma-Aldrich, cat. no. I8896)

- Tris (Carl Roth, cat. no. 5429)
- Tween-20 (Sigma-Aldrich, cat. no. P7949)
- Thermoprime 2× ReddyMix PCR Master Mix (cat. no. AB0575DCLDB)

Consumables

- Corning Costar 100 mm TC-Treated Culture Dishes (Sigma-Aldrich, cat. no. 430167)
- Cellstar cell culture 6-well plates (Greiner Bio-One, cat. no. 657160)
- Corning Costar cell culture plates 96-well flat bottom (Sigma-Aldrich, cat. no. 3598)
- Corning Costar cell culture plates 96-well round bottom (Sigma-Aldrich, cat. no. 3799)
- 96-well PCR plates (Thermo Scientific, cat. no. AB-0900)
- Adhesive PCR Plate seals (Biozym, cat. no. 600208)
- 20 µm sterile Cell Trics filters (Partec, cat. no. 04-004-2325)
- 0.5 - 10 µl / 10 - 100 µl / 100 - 1’000 ml pipettes (e.g. Eppendorf Research plus)
- Corning Costar Stripette serological pipettes, 5 ml (e.g. Sigma-Aldrich)
- Microcentrifuge tubes, transparent, 1.5 ml (e.g. Brand)

Equipment

- 12-Channel Pipette (e.g. Eppendorf Research plus)
- Repeater Pipette (e.g. Eppendorf Multipette M4)
- Manifold for 96 hole plate (e.g. Thomas Scientific 7691R60)
- Nucleofector 2b Device (Lonza, cat. no. AAB-1001)
- Cell culture incubator (at 36.5 °C and 10 % CO_2; e.g Thermo Scientific)
- 96-well PCR cycler (e.g. Bio-Rad)
- Incubator at 55°C (e.g Thermo Scientific)
- Inverted tissue culture light microscope (e.g. Carl Zeiss Axiovert 25)
- Automated cell counter (e.g. Countess II FL Automated Cell Counter)
- Cell sorter (e.g. BD FACS Aria III)

Reagent Setup

MDCK medium

DMEM is supplemented with 10 % (vol / vol) FBS and 1 % (vol / vol) Penicillin-Streptomycin. Store the medium at 4 °C and use it within 2 months.

mIMCD3 medium

DMEM : Ham's F12 is supplemented with 10 % (vol / vol) FBS and 1 % (vol / vol) Penicillin-Streptomycin. Store the medium at 4 °C and use it within 2 months.

mESC medium

500 ml DMEM is supplemented with 15 % (vol / vol) ESC certified FBS, 1 % (vol / vol) Glutamine 100×, 1 % (vol / vol) Penicillin-Streptomycin, 3.5 µl 2-Mercaptoethanol, and 10^6 U of LIF. Store the medium at 4 °C and use it within 2 months.

Freezing medium

Supplement MDCK or mIMCD3 medium with 10 % FBS and 10 % DMSO. Use immediately.

Lysis buffer

10 ml 1 M KCl; 300 µl 1 M $MgCl_2$; 2 ml 1 M Tris pH 8.5; 900 µl Igepal CA-630; 900 µl Tween-20; make up to 200 ml with H_2O; re-adjust the pH value to 8.5 if necessary; aliquot into 10 ml aliquots; freeze at -20 °C.

General Considerations

Most problematic for the development of genome editing-based cell lines are efficacy, specificity, and screenability. Attached protocols discuss the critical steps to efficiently generate arbitrary genomic alterations, identify correctly targeted clones, and establish genetically modified cell lines within a short time-frame of ~10 weeks.

Genome editing technology

Genome editing is based on the induction of genomic double strand breaks (iDSBs) and subsequent exploitation of the cellular DNA repair mechanisms: non-homologous end joining (NHEJ) and homology directed repair (HDR) (4, 9). Here, we used TALE- and CRISPR-based nucleases to induce DNA double strand breaks (**Supplementary Figure 1a,b**) (4, 6, 7, 9). Genome editing technologies are still in an innovative state of flux with novel adaptations being published on a frequent basis. For the development of novel *in vitro* models, it is important to note that selection of designer nucleases is just one step in a complex workflow. Novel technologies, including new Cas9 variants or genome editing binding site requirements, may be incorporated without changing the subsequent screening and selection procedures (**Figure 2a**).

Genome editing in cell culture

Both, TALENs and the CRISPR system, are highly effective genome editing tools in MDCK, mIMCD3, and mES cells. To maximize specificity, we use split genome editing tools, i.e. binding of two TALENs (dimeric Fok1) (5) or two sgRNAs (Cas9n) (12) is required to induce DSBs (**Supplementary Figure 1a,b**) (4, 9). The recommended distance between two TALENs for one DSB is 13 bp, for two sgRNAs in combination with Cas9n 10-31 bp (4, 9). In our experience, split genome editing tools are less efficient in generating heterozygous mutations compared to their non-split alternatives (~5 % vs. ~20 %), but the probability of off-target effects is significantly lower (4, 9). The impossibility of outcrossing putative off-target effects in cell culture mandates high specificity over efficiency for most applications. Low efficiency can be alleviated by scalability of cell culture models.

MDCK, mIMCD3, and mES cells

To guarantee defined experimental conditions, it is advised to use only well-characterized, low passage MDCK, mIMCD3, and mES cells of documented origin. Given the heterogeneity of MDCK and mIMCD3 cells, it is useful to establish a number of clonal wild-type cell lines as isogenic starting material for genome editing as well as isogenic controls for subsequent experiments (2, 10). When genome editing is performed to generate novel mouse strains, mESCs used should be proven for their potency to form high chimerism and to undergo high-frequency germline transmission. Cultured cells should always be treated with utmost care and according to

established standards (1, 3, 10). mESCs require specific handling to maintain potency for germline transmission, e.g. maintenance of ESCs on mitotically inactivated murine embryonic fibroblasts (MEFs) and specific splitting regimes. Unless otherwise stated, ESC culture of CCE ESCs is performed as described previously (1).

Passaging of MDCK and mIMCD3 cells 1 : 10 in 100 mm (35 mm) dishes:
MDCK and mIMCD3 cells are cultivated as adherent monolayers. Cell lines are maintained in a humidified 10 % CO_2 incubator at 36.5 °C. Cells have to be passaged upon confluency, usually 2 - 3 times per week.

1. Remove medium;
2. Wash cells with 10 ml (2 ml) pre-warmed (37 °C) PBS;
3. Add 1 ml (0.25 ml) 0.25 % Trypsin-EDTA;
4. Incubate at 36.5 °C until cells detach (5 - 15 min);
5. Prepare new dishes with 10 ml (2 ml) fresh medium;
6. After cells have detached, add 9 ml (1.75 ml) fresh medium to inactivate trypsin;
7. Homogenize cell suspension by pipetting with a 5 ml serological strippette (10 × up-and-down);
8. Transfer 1 ml cell suspension into every new dish;
9. Move dishes horizontally to mix.

Genome editing in renal epithelial cells and mESCs

Here we describe an efficient workflow to employ TALENs and the CRISPR system in MDCK, mIMCD3 and mES cells allowing for the introduction, alteration, and/or removal of genomic sequences (**Figure 2a,b**). Removal of genomic sequences takes advantage of NHEJ; introduction or alteration of genomic sequences is based on HDR. For loss-of-gene function models, we suggest deleting respective genes in total to generate genomic, mRNA, and protein null cells (**Supplementary Figure 1c**). The reason being that, for many genes, the absence of functional data impairs evaluation of deleterious effects of missense, nonsense, splice site, and frameshift mutations. Testing for unspecific effects due to concomitant deletion of regulatory regions or small RNA binding sites, on the other hand, is straight forward by evaluating observed phenotypes in rescue experiments, i.e. by re-introduction of respective cDNAs into deficient cells.

Procedure

1. Defining genome editing target-sites

General design and target-site identification of CRISPR sgRNAs and TALENs have been described previously (4, 9). Useful tools to automate design of genome editing constructs include: http://chopchop.cbu.uib.no and http://www.e-talen.org/E-TALEN. Selection of specific binding sites is, of course, highly project-dependent. It has to be stressed that TALEN- and CRISPR-mediated genome editing are local events (**Supplementary Figure 1a,b**). DSBs will be induced at the binding site, causing local NHEJ or HDR. Consequently, HDR-mediated gene targeting efficiency decreases significantly when the cut site is > 30 bp from the proximal end of the repair template (8, 11). Because genome editing efficacy frequently depends on the genomic locus, we regularly perform CRISPR and TALEN approaches in parallel, until one approach has proven effective in targeting the respective gene of interest. Irrespective of planned genome alteration, screenability of cells has to be taken into account. For this, it has proven useful to design paired TALENs and CRISPR sgRNAs that in combination result in genomic deletions (**Supplementary Figure 1a-c**). Deletions can be detected easily using PCR and subsequent gel electrophoresis. The small sequence variants usually observed when single editing tool are used, on the other hand, require additional sample processing compromising identifiability. Furthermore, by placing both, or in case of whole gene-deletions, at least the 5'-binding construct in protein coding exons, deleterious NHEJ-mediated InDels may still be recovered even when full deletions are ineffective at maximizing the number of potentially useful alleles.

1a. HDR template - targeting vector design

Genome editing tools taking advantage of HDR and NHEJ are similar. The main difference is the addition of an HDR template to the genome editing constructs. This targeting vector consists of two flanking homology regions and sequence elements that lie within the homology arms (**Supplementary Figure 1d**). Sequence elements may include reporter constructs, epitope-tags, cDNAs, and selection cassettes that indicate integration into the genome. For HDR approaches 110 bp homology arms are effective in MDCK and mIMCD3 cells (**Supplementary Figure 1d**). For targeting in mESCs, longer homology regions of 500 bp were chosen, but could be reduced in size similarly. From a technical point of view, this simplifies targeting vector design significantly. Many manufacturers provide oligonucleotides of up to 120 bp by default, i.e. targeting vectors may be designed *in silico* and assembled from 2-5 HPLC-cleaned oligonucleotides *in vitro*. Alternatively, entire targeting constructs can be obtained from commercial DNA synthesis providers. To avoid re-editing of desired alleles, binding sites of genome editing constructs in targeting vectors, including the CRISPR PAM sequence, should be removed by mutation of respective sites using species optimized, non-coding sequence alterations (**Supplementary Figure 1d**) (8). A neomycin

resistance cassette for positive selection of MDCK and mIMCD3 clones may be integrated into the targeting vector (**Supplementary Figure 1d**). MDCK and mIMCD3 cells are sensitive to geniticin, 810 units / ml for 10 days and 7 days respectively. However, in our hands the rate of true positive clones in geniticin selected cell culture does not differ from non-selected cells significantly. Parallel introduction of a negative selection HSVtk cassette (2 µg / ml) seems to reduce false positive clones (**Supplementary Figure 1d**). A general disadvantage of selection cassettes is the artificial incorporation of exogenic material into the genome questioning the moderate advantage in MDCK and mIMCD3 cells. All ADPKD cell lines described here have been generated without selection. In contrast, for mESCs that show significantly lower overall transfection rates, the introduction of a positive selection cassette into the targeting vector is strongly advised.

2. Testing of screening strategy

Screening of genome-edited cells for deletions or correct integration by homologous recombination is performed by PCR. Robust screening PCRs are key and emphasis should be put on reliable PCR conditions (**Supplementary Figure 1c**). All PCRs to detect genomic wild-type configurations should be optimized in wild-type cells beforehand. PCRs to detect genome-edited sequences should be tested by a first round of construct transfection and bulk analysis of resulting cells (see below). *A priori* design of two sets of oligonucleotides per PCR has proven valuable. Tools like Primer3Plus (http://primer3plus.com/cgi-bin/dev/primer3plus.cgi) can automate homogenous PCR oligonucleotide design (PCR product size 200-500 bp; T_m ~60 °C).

3. Transfection of MDCK, mIMCD3, and mES cells

Transfect low-passage MDCK and mIMCD3 cells at 80 % confluency with the respective genome editing constructs according to the nucleofection protocol for these cell lines. Deviating from the manufacturer's protocol, input of 1×10^6 cells per transfection ensures better cell survival for genome editing. A total of 5 µg DNA may be transfected per nucleofection. Transfect genome editing constructs in equal amounts, e.g. 1.25 µg per TALEN when using four TALEN constructs, or 1 µg per genome editing construct when using four sgRNAs and a linearized targeting vector. Repeated transfection of cells three days after first transfection increases the proportion of targeted cells and total time of expression significantly (**Figure 2c**). For targeting of ES cells 5×10^6 cells are nucleofected with 1.25 µg per TALEN-/CRISPR-construct and 5 µg of linearized targeting vector. Prior removal of MEF feeder cells is not required, despite recommendation by the manufacturer. mESCs are nucleofected twice with a time interval of 24 h. After second transfection, mESCs are plated at a density of 1-5 × 10^5 cells on 10 cm dishes with MEF feeder cells. Positive selection is initiated 24 h after plating. Selection-resistant clones can be manually picked into 96-well format after ~8 days.

4. Testing of genome editing efficacy

Two days after single or double transfection, efficacy of genome editing constructs may be evaluated. Individual binding sites for genome editing construct should be assessed for evidence of NHEJ, e.g. by subcloning and sequencing of PCR products, or high resolution melting analysis. Functional TALENs and CRISPR induce a high rate of local mutations (~20 %). Genome editing-mediated target alleles should be validated by PCR and sequencing. Negative PCR results may be due to ineffective PCR amplification of target alleles or ineffective genome editing constructs. If two independent PCR screening strategies yield negative results and sequencing of wild-type alleles is inconspicuous, ineffectiveness of genome editing constructs can be assumed. Proceed only if genome editing constructs are effective in generating the desired allele. Otherwise, devise a new genome editing strategy (**Figure 2a**).

Lysis of MDCK and mIMCD3 cells in 35 mm dishes:

1. Prepare lysis buffer by adding 10 µl proteinase K (10 mg / ml) to 1 ml crude lysis buffer;
2. Remove cell culture medium;
3. Wash cells twice with 150 µl PBS;
4. Add 1 ml lysis buffer;
5. Incubate 1.5 h at 55 °C;
6. Homogenize lysate by pipetting 5 × up-and-down;
7. Transfer lysate into 1.5 ml tube;
8. Incubate 10 min at 95 °C to inactivate proteinase K;
9. Store lysates at -20 °C.

15 µl PCRs for screening:

1. Provide 1 µl cell lysate;
2. Add 1 µl oligonucleotide mix (10 µM each);
3. Add 5.5 µl H_2O;
4. Add 7.5 µl 2× ReddyMix PCR Master Mix;
5. PCR conditions:
    1. 95 °C 2 min;
    2. 95 °C 25 sec;
    3. 60 °C 35 sec;
    4. 72 °C 1 min / 1’000 bp;
    5. go to 2 for 39×;
    6. 72 °C 5 min;
    7. Hold 8 °C;
6. Run 1 % agarose gel to assess PCR products.

5. Clonal expansion of targeted cells

MDCK and mIMCD3 cells can be sorted as single cells three days after the second round of transfection. We use a BD FACS Aria III cell sorter to singularize cells. Average probability for complex genome editing events is ~5 %, i.e. 1 in 20 cells will carry a heterozygous mutation and 1 in 400 will be homozygous (**Figure 2e**). Screening of clones is labour-intensive. Manual handling of more than 600 clones in parallel has proven ineffective. Subsequent screening is recommended. For MDCK cells inoculation of six 96-well plates, for less clonogenic mIMCD3 cells inoculation of twelve 96-well plates is advised to ensure survival of sufficient clones for screening (**Figure 2d**). mESCs were plated at relatively low density and grown under positive selection to generate separated ESC clones that can be manually transferred into a 96-well format.

Individualization of MDCK and mIMCD3 cells from a 35 mm dish for automated cell sorting:

1. Remove medium;
2. Wash cells with 2 ml pre-warmed (37 °C) PBS;
3. Add 500 µl 0.25 % Trypsin-EDTA;
4. Incubate at 36.5 °C until cells detach (5-15 min);
5. Control detachment by microscope;
6. After cells have detached, add 1 ml fresh medium to inactivate trypsin;
7. Individualize cells by pipetting vertically (pressing the pipette tip on the dish) with a 1 ml pipette (20 × up-and-down);
8. Filter cells into a 15 ml tube;
9. Immediately proceed to cell sorting.

Picking of mESCs into 96-well format:

1. Prepare 96-well plates with a layer of MEF-feeder cells in mESC medium;
2. Add 25 µl 1x trypsin solution per well into 96-well round bottom plates;
3. Wash mESCs grown on 10 cm dish with 2 ml pre-warmed (36.5 °C) PBS;
4. Transfer one ESC colony to each of trypsin-containing 96-wells using a micropipette;
5. Incubate plate for 5 min in humidified CO_2 incubator at 36.5 °C;
6. Add 75 µl mESC medium per well;
7. Produce a single cell suspension by trituration;
8. Transfer 50 µl of mESC suspensions onto feeder layers;
9. Transfer 25 µl of mESC suspension into 96-well PCR plate for later processing for genotyping (as described below);
10. Repeat previous three steps for remaining rows;

11. Repeat trypsinization, trituration and seeding steps until desired number of colonies have been picked.

6. Cell expansion in 96-well format

After transfer into 96-well format MDCK cells will need ~2 weeks to become confluent, mIMCD3 cells will need ~4 weeks, and mESC colonies will require further processing after 2-4 days. Proliferation of MDCK and mIMCD3 cells from single cell to confluent epithelium is highly variable after single cell sorting. Cells should be monitored closely to assess confluency, survival, and morphology. If there is no indication that the target allele impacts proliferation, cells may be split after ~60 % of wells have reached confluency. If less than 50 % of wells are occupied, consolidation of plates may decrease subsequent workload and cost of materials.

Passaging of MDCK and mIMCD3 cells 1 : 6 in 96-well format:

While screening for positive clones, MDCK and mIMCD3 cells have to be maintained in 96-well format. To guarantee survival cells must be passaged once per week 1 : 6. Prepare one plate for maintenance and one plate for PCR-based screening for targeted alleles.

1. Remove medium;
2. Wash cells with 150 µl pre-warmed (37 °C) PBS;
3. Add 50 µl 0.25 % Trypsin-EDTA;
4. Incubate at 36.5 °C until cells detach (5-15 min);
5. Control detachment by microscope;
6. While waiting for detachment, prepare new 96-well plates with 125 µl fresh medium per well;
7. After cells have detached, add 100 µl fresh medium to inactivate trypsin;
8. Homogenize cell suspension by pipetting 5 × up-and-down;
9. Transfer 25 µl cell suspension into every new well;
10. Move 96-well plates horizontally to mix.

7. Screening for targeted alleles

Continuous maintenance of 96-well plates is time-consuming. Hence, timely analysis of targeted cells is highly desirable. Two days after the first passaging of clones in 96-well format, survival of clones can be assessed and PCR-based screening may be started.

In-96-well plate lysis of MDCK and mIMCD3 cells:

1. Prepare lysis buffer by adding 100 µl proteinase K (10 mg / ml) to 10 ml crude lysis buffer;
2. Remove cell culture medium;
3. Wash cells twice with 150 µl PBS;
4. Add 100 µl lysis buffer;

5. Incubate 1.5 h at 55 °C;
6. Homogenize lysate by pipetting 5 × up-and-down;
7. Transfer lysate from 96-well flat bottom plate into 96-well PCR plate;
8. Incubate 10 min at 95 °C to inactivate proteinase K;
9. Immediately proceed to PCR screening or store lysates at -20 °C.

In-96-well plate lysis of mESCs after picking of mESC colonies:

1. Prepare lysis buffer by adding 100 µl proteinase K (10 mg / ml) to 10 ml crude lysis buffer;
2. Spin down mESCs in 25 µl trypsin/mESC-medium directly after picking into 96-well plate (as described above: *Picking of mESCs into 96-well format, step 9.*);
3. Carefully remove supernatant but not ESCs from each well;
4. Add 100 µl lysis buffer;
5. Incubate for 1.5 h at 55 °C;
6. Incubate 10 min at 95 °C to inactivate proteinase K;
7. Immediately proceed to PCR screening or store lysates at -20 °C.

96-well PCR for identification of correctly targeted alleles:

1. Perform screening PCRs using previously tested conditions;
2. Analyse PCR products by agarose gel-electrophoresis.

7a. PCR-screening for targeted deletions

To identify targeted deletions two PCR approaches should be designed: one for the wild-type locus; and one for the deleted allele. Ideally, both configurations should be detected with one identical primer binding-site, so that PCR products for the wild-type and deleted allele can be generated in the same PCR reaction using three oligonucleotides in total (**Supplementary Figure 1c**). In cases where two independent PCRs have to be performed, all clones should be screened for the presence of a wild-type allele. Presence of a wild-type band indicates good DNA quality; positive clones are wild-type or heterozygous. All clones (if heterozygous clones are desired) or wild-type negative clones only may be screened for the deletion allele. If screening only the negative subset of clones, a positive control PCR should be performed for the assessment of DNA quality. Clones negative for the wild-type allele and positive for the targeted allele are most likely homozygous for the deletion (**Supplementary Figure 1c**). However, unintended complex genomic rearrangements should be tested by DNA-independent validation of targeted alleles.

7b. PCR-screening for homologous recombination

Screening for target vector integrations by HDR is performed similarly to genome editing-mediated deletions. PCRs should be designed to detect wild-type and target alleles. For the latter, one

oligonucleotide-binding site should lie outside of the targeting vector homology-arm, and the second within a newly introduced sequence element (**Supplementary Figure 1e,f**). If possible the locus-specific oligonucleotide should be used in wild-type and target PCR. Screening based on PCR size difference, on the other hand, may be biased due to preferential amplification of small PCR-fragments. Correct target integration by HDR requires validation by sequencing. Imperfect, unilateral integration is observed regularly in all cell lines tested. Integrity of wild-type alleles in heterozygously targeted clones should be validated by sequencing, because minor NHEJ-mediated mutations will usually not affect PCR results.

8. Clone Expansion

Clones of interest are transferred from 96-well format into 6-well format. Original 96-well plates may be frozen as temporary backup at -80 °C with good cell survival. Initially, MDCK cells will become confluent ~7 days after transfer, mIMCD3 cells after ~12 days.

Temporary freezing of MDCK and mIMCD3 cells in 96-well format:

1. Remove medium;
2. Wash cells with 150 µl pre-warmed (37 °C) PBS;
3. Add 150 µl of freezing medium;
4. Cool cells on ice for 30 min;
5. Put plate into pre-cooled thick styrofoam container;
6. Place container into -80 °C freezer.

Transfer of individual clones from 96-well plates into 6-well format:

Although only some clones per plate may be of interest, multichannel pipette-based handling of the whole 96-well plate is most efficient.

1. Remove medium;
2. Wash cells with 150 µl pre-warmed (37 °C) PBS;
3. Add 50 µl 0.25 % Trypsin-EDTA;
4. Incubate at 36.5 °C until cells detach (5-15 min);
5. Control detachment by microscope;
6. While waiting for detachment, prepare 6-well with 2 ml fresh medium per well;
7. After cells have detached, add 100 µl fresh medium to inactivate trypsin;
8. Homogenize cell suspension by pipetting 5 × up-and-down;
9. Transfer 125 µl cell suspension into every new well;
10. Move 6-well plates horizontally to mix;
11. To maintain the 96-well plate as backup add 125 µl fresh medium / well.

9. Validation of targeted clones

From expanded cell clones newly prepared genomic DNA should be evaluated by PCR to exclude sample mix-up. If clones appear homozygous, allelic PCR products should be subcloned and expanded in *E. coli*. Screening of at least six *E. coli* colonies can surface minor allelic differences or confirm homozygosity. Alternatively, high resolution melting analysis or similar methods may be used to evaluate wild-type PCR homogeneity. For TALENs we have observed very homogenous genome editing results, e.g. both deletion alleles are identical; the CRISPR system, on the other hand, seems to produce less predictable results. Low passage clones should be frozen to backup cell culture.

10. DNA independent validation of target alleles

Once cells are cultured in ≥6-well format, RT-PCR and protein-based methods may be used to validate target allele properties, e.g. absence of mRNA after genomic deletion or presence of epitope-fused protein after genomic knock-in.

11. Re-transfection of heterozygous clones

Heterozygous clones carrying sequence-validated wild-type and targeted alleles may be used for re-targeting with genome editing constructs. Proceedings are similar to wild-type cells, but scale may be adapted to the observed frequency of respective genome editing events. Usually, ~5 %, i.e. 1 in 20 cells, will carry homozygous mutations after re-transfection.

Clonal artefacts

It should be attempted to isolate three homozygous and three heterozygous knock-out or HDR clones. Genome-editing in MDCK and mIMCD3 cells is based on single cell culture and therefore prone to clonal artifacts.

Supplementary Figures

Supplementary Figure 1 | Practical guide to genome editing in MDCK and mIMCD3 cells.

Binding of (**a**) TALENs and (**b**) the CRISPR system to the mouse *Pkd1* locus. DNA strand breaks are induced (red arrows) and cellular DNA repair systems activated, i.e. non-homologous end joining (NHEJ) and homology directed repair (HDR). PAM, protospacer adjacent motif. (**c**) PCR screening strategy for the deletion of *Pkd1* in mIMCD3 cells. PCR using oligonucleotides 1 + 2 yields a 582 bp product when the *Pkd1* wild-type locus is preserved. PCR with the oligonucleotides 2 + 3 will produce a 411 bp band only if *Pkd1* has been deleted. Upon *Pkd1* deletion the wild-type PCR will turn negative because the binding site for oligonucleotide 1 has been deleted. Oligonucleotides: 1 = mPkd1_1; 2 = mPkd1_2; and, 3 = mPkd1_3. (**d**) Schematic of targeting vector. 110 bp recombination arms have proven effective. Targeting vectors may contain selection cassettes (neomycin and/or HSVtk) and epitope tags (Flag). TALEN and CRISPR sgRNA binding sites need to be mutated in the targeting vectors to render applied genome editing constructs ineffective on target alleles. (**e**) PCR screening strategy for a 72 bp triple-Flag knock-in into the mIMCD3 *Pkd1* genomic locus. PCR using oligonucleotides 1 + 2 will show a size shift upon knock-in. However, size-shift-based PCRs may be prone to inhomogeneous amplification of alleles. More sensitive are PCRs based on HDR-mediated introduction of oligonucleotide binding sites, i.e. PCRs with oligonucleotides 1 + 3 and 4 + 2. Oligonucleotides: 1 = mPkd1-Flag_1; 2 = mPkd1-Flag_2; 3 = mPkd1-Flag_3; and, 4 = mPkd1-Flag_4. (**f**) Similarly, PCR screening strategy for a 90 bp double-V5 knock-in into the mIMCD3 *Pkd1* genomic locus. Oligonucleotides: 1 = mPkd1-V5_1; 2 = mPkd1-V5_2; and, 3 = mPkd1-V5_3.

Supplementary Figure 2 | Targeting strategy for the Generation of a Lama5^{GFP} allele.

(**a**) TALENs were designed to induce double strand breaks within the last exon (#79) of the *Lama5* locus. Dual targeting with TALENs (indicated by red bars) generated a NHEJ-mediated deletion of 220 bp, when used without a targeting vector. (**b**) The targeting vector for the genomic integration of GFP cDNA is designed to create a C-terminal GFP-fusion to the LAMA5 protein. It contains 500 bp of genomic sequences 5' to the TGA stop-codon (including exons 78 and 79), a 30 bp linker (alanin-serin-repeats), a cDNA encoding eGFP, a bGH Poly-A signal, a loxP-flanked phosphoglycerate kinase I (PGK) promoter driven neomycin resistance cassette (Neo^r), and 500 bp of genomic sequences 3' to the TGA stop-codon of *Lama5*. To prevent TALEN-induced double strand breaks of the targeting vector, TALEN-binding sequences were point-mutated in the targeting vector. (**c**) Electoporation of two TALENs in combination with the targeting vector, followed by G418 (Neomycin) selection generated the *Lama5^{GFP} Neo^r* allele, and (**d**) the final *Lama5^{GFP}* allele after *Cre*-mediated deletion of the Neo^r cassette.

Supplementary Tables

Supplementary Table 1 | Cell lines. ADPKD cell lines will be available through the Baltimore PKD Research and Clinical Core Center (http://baltimorepkd.org). Genomic positions are based on mouse reference genome GRCm38 and dog CanFam3.1.

Supplementary Table 2 | TALEN and sgRNA binding sites.

Supplementary Table 3 | Genotyping of mIMCD3 cell lines.

Supplementary Table 4 | Genotyping of MDCK cell lines.

Supplementary Table 5 | Oligonucleotides for RT-PCR.

Supplementary Table 6 | Polycystin-1 nano-LC-MS/MS peptide data.

References

1. **Behringer R**. *Manipulating the mouse embryo : a laboratory manual*. Cold Spring Harbor, New York: Cold Spring Harbor Laboratory Press, 2014, p. xxii, 814 pages.
2. **Dukes JD, Whitley P, and Chalmers AD**. The MDCK variety pack: choosing the right strain. *BMC Cell Biol* 12: 43, 2011.
3. **Gaush CR, Hard WL, and Smith TF**. Characterization of an established line of canine kidney cells (MDCK). *Proc Soc Exp Biol Med* 122: 931-935, 1966.
4. **Joung JK, and Sander JD**. TALENs: a widely applicable technology for targeted genome editing. *Nature reviews Molecular cell biology* 14: 49-55, 2013.
5. **Miller JC, Tan S, Qiao G, Barlow KA, Wang J, Xia DF, Meng X, Paschon DE, Leung E, Hinkley SJ, Dulay GP, Hua KL, Ankoudinova I, Cost GJ, Urnov FD, Zhang HS, Holmes MC, Zhang L, Gregory PD, and Rebar EJ**. A TALE nuclease architecture for efficient genome editing. *Nature biotechnology* 29: 143-148, 2011.
6. **Morbitzer R, Elsaesser J, Hausner J, and Lahaye T**. Assembly of custom TALE-type DNA binding domains by modular cloning. *Nucleic acids research* 39: 5790-5799, 2011.
7. **Mussolino C, Morbitzer R, Lutge F, Dannemann N, Lahaye T, and Cathomen T**. A novel TALE nuclease scaffold enables high genome editing activity in combination with low toxicity. *Nucleic acids research* 39: 9283-9293, 2011.
8. **Paquet D, Kwart D, Chen A, Sproul A, Jacob S, Teo S, Olsen KM, Gregg A, Noggle S, and Tessier-Lavigne M**. Efficient introduction of specific homozygous and heterozygous mutations using CRISPR/Cas9. *Nature* 533: 125-129, 2016.
9. **Ran FA, Hsu PD, Wright J, Agarwala V, Scott DA, and Zhang F**. Genome engineering using the CRISPR-Cas9 system. *Nat Protoc* 8: 2281-2308, 2013.
10. **Rauchman MI, Nigam SK, Delpire E, and Gullans SR**. An osmotically tolerant inner medullary collecting duct cell line from an SV40 transgenic mouse. *Am J Physiol* 265: F416-424, 1993.
11. **Rivera-Torres N, Strouse B, Bialk P, Niamat RA, and Kmiec EB**. The position of DNA cleavage by TALENs and cell synchronization influences the frequency of gene editing directed by single-stranded oligonucleotides. *PLoS One* 9: e96483, 2014.
12. **Shen B, Zhang W, Zhang J, Zhou J, Wang J, Chen L, Wang L, Hodgkins A, Iyer V, Huang X, and Skarnes WC**. Efficient genome modification by CRISPR-Cas9 nickase with minimal off-target effects. *Nature methods* 11: 399-402, 2014.
